# A Non-Destructive and Direction-Insensitive Method Using a Strain Sensor and Two Single Axis Angle Sensors for Evaluating Corn Stalk Lodging Resistance

**DOI:** 10.3390/s18061852

**Published:** 2018-06-06

**Authors:** Qingqian Guo, Ruipeng Chen, Xiaoquan Sun, Min Jiang, Haifeng Sun, Shun Wang, Liuzheng Ma, Yatao Yang, Jiandong Hu

**Affiliations:** 1 Collage of Mechanical and Electrical Engineering, Henan Agricultural University, Zhengzhou 450002, China; guoqingqian369@gmail.com (Q.G.); 230179522@seu.edu.cn (R.C.); hnndsunhaifeng@gmail.com (H.S.); hnndwangshun@gmail.com (S.W.); mlz0124@126.com (L.M.); yataoyang@yeah.net (Y.Y.); 2 State Key Laboratory of Wheat and Maize Crop Science, Zhengzhou 450002, China; 3 Henan Institute of Metrology, Zhengzhou 450008, China; xiaoquan_sun@sina.com; 4 College of Life Sciences, Henan Agricultural University, Zhengzhou 450002, China; jmhu@henau.edu.cn

**Keywords:** strain sensor, single axis angle sensor, corn stalk, equivalent force, lodging rate

## Abstract

Corn stalk lodging is caused by different factors, including severe wind storms, stalk cannibalization, and stalk rots, and it leads to yield loss. Determining how to rapidly evaluate corn lodging resistance will assist scientists in the field of crop breeding to understand the contributing factors in managing the moisture, chemical fertilizer, and weather conditions for corn growing. This study proposes a non-destructive and direction-insensitive method, using a strain sensor and two single axis angle sensors to measure the corn stalk lodging resistance in the field. An equivalent force whose direction is perpendicular to the stalk is utilized to evaluate the corn lodging properties when a pull force is applied on the corn stalk. A novel measurement device is designed to obtain the equivalent force with the coefficient of variation (CV) of 4.85%. Five corn varieties with two different planting densities are arranged to conduct the experiment using the novel measurement device. The experimental results show that the maximum equivalent force could reach up to 44 N. A strong relationship with the square of the correlation coefficient of 0.88 was obtained between the maximum equivalent forces and the corn field’s stalk lodging rates. Moreover, the stalk lodging angles corresponding to the different pull forces over a measurement time of 20 s shift monotonically along the equivalent forces. Thus, the non-destructive and direction-insensitive method is an excellent tool for rapid analysis of stalk lodging resistance in corn, providing critical information on in-situ lodging dynamics.

## 1. Introduction

Stalk lodging resistance has always been an important aspect of plant quality in corn. However, there is little accurate information on corn stalk lodging resistance due to experimental restrictions [1,2]. Stalk lodging is the breakage of the stalk at or below the ear, which may result in a loss in the yield at harvest [3,4]. The tissue of the corn stalk is destroyed in the process of stalk lodging, which affects water and nutrient transportation and photosynthetic efficiency [5,6,7]. It is generally known that lodging is largely influenced by the strength of the stalk, which is determined by the diameter of the internodes, the internodes’ distance, and plant height [8,9]. Selective breeding approaches were traditionally applied to improve stalk lodging properties; such approaches relied on counting the number of stalks that had already broken or lodged below the ear at harvest [10,11]. A severe wind storm may nearly blow down an entire field of corn and leave corn seldom standing, and it is impossible to distinguish stronger corn varieties from weaker varieties [12]. Currently, the most effective method to evaluate the property of stalk lodging is a laboratory wind tunnel experiment. An intuitive proportional relationship can be obtained between stalk lodging resistances and wind speed in the laboratory, using wind tunnel experiments [13,14,15]. Although wind tunnel experiments can be used to determine the stalk lodging resistance in corn, they are not convenient for the in-situ quantitative analysis of stalk lodging properties. The strength of corn stalks was appraised by hydraulic machinery applied to crush a stalk, where both the stalk crushing strength (SCS) and rind penetrometer resistance (RPR) are commonly used to evaluate the mechanical characteristics of corn stalks [16,17]. However, mechanical measurements to test stalk strength are typically destructive and time-consuming methodologies (involving physically breaking or crushing the stalk). Thus, a stalk hardness meter was developed to evaluate stalk lodging properties [18,19,20]. Correlation analyses revealed that stalk flexural stiffness predicted 81% of the variation in stalk strength, whereas rind penetration resistance only accounted for 18% of the variation in stalk strength [21,22]. Moreover, the rind penetration resistance of stalks cannot fully reflect the influence on stalk lodging caused by the natural wind, and the measuring results from the stalk hardness meter are greatly dependent on the probe shapes [23]. Scientists also put forward a method to evaluate stalk lodging properties by measuring a force directly exerted on the corn stalk. However, the measurement results were largely subject to the direction of pull force, and many aspects of this method still needed to be improved [24,25]. More rapid gains in corn stalk lodging resistance could be achieved by developing testing methodologies that can predict stalk lodging properties in the absence of stalk lodging related weather events [26]. In order to solve these problems, an innovative method is proposed in this paper to quantitatively determine stalk lodging properties during the stalk lodging process, where an equivalent force is immune to the direction of pull force was used. The equivalent force can be used to evaluate the corn stalk lodging properties. The obtained data can be provided for agricultural scientists to infer the corn stalk lodging process and to effectively evaluate the affective factors.

## 2. Materials and Methods

### 2.1. Plant Materials

**Figure 1:**
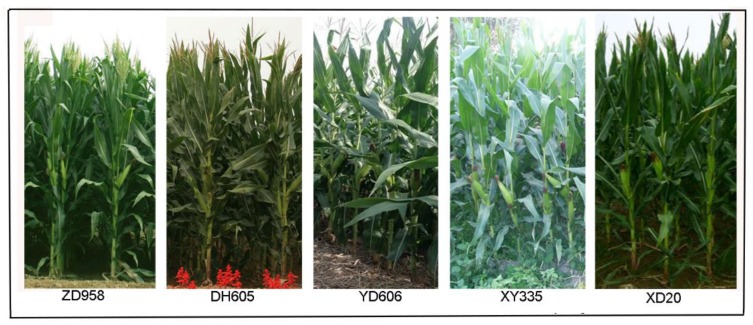
Five varieties of corn grown in the Science and Education Farm of Henan Agriculture University, Zhengzhou.

Five varieties of corn labeled Zhengdan958 (ZD958), Xianyu335 (XY335), Yudan606 (YD606), Xundan20 (XD20), and Denghai605 (DH605) were available for these experiments (Figure 1). The measured agronomic traits are indicated in Table 1. One-way analysis of variance (ANOVA) was used to determine whether there were any statistically significant differences among the treatment means, which were obtained from five corn varieties with two planting densities. Different varieties of corn were grown in the Science and Education Farm of Henan Agriculture University, Zhengzhou (113.66° E, 34.76° N). Five varieties of corn were provided by the Henan Academy of Agricultural Sciences, Tieling Xianfeng Seed Research Co., Ltd. (Tieling, China), Henan Agricultural University, the Agricultural Science Research Institute of Xunxian, and Henan Shandong Denghai Seed Industry Co, Ltd. (Zhengzhou, China), respectively. Planting areas were used with a plant distribution of two planting densities, 60,000 plants/hm^2^ and 75,000 plants/hm^2^. Each planting area was 6 m long with 6 rows 0.6 m wide. The same fertilization management was utilized during two periods of corn growth (organic matter of 10.8 g/kg, total nitrogen of 0.93 g/kg, available phosphorus of 25 mg/kg, and available potassium of 125 mg/kg). The corn stalks in the stage of silking (20 days before maturity) were arranged for these experiments from June to October in 2016 and 2017, respectively. 

### 2.2. Measurement System Design

**Figure 2:**
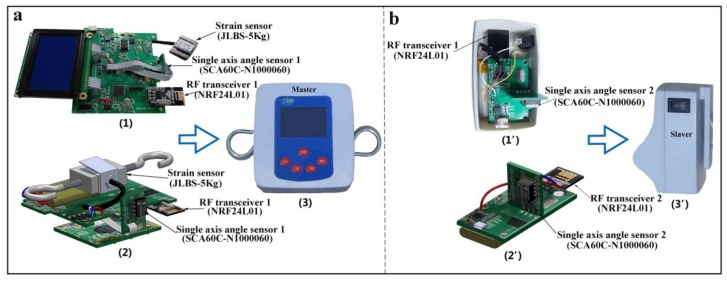
Photos of the measurement device and the corresponding electronic components. (**a**) The electronic components of the Master unit; (**b**) the electronic components of the Slaver unit.

The measurement device was composed of separate circuit blocks marked with Master unit and Slaver unit, and the photos of the corresponding electronic components are shown in Figure 2. From Figure 2a, the Master unit mainly consisted of a strain sensor (JLBS-5Kg), a single axis angle sensor (SCA60C-N1000060) perpendicular to the circuit board of the Master unit to keep the initial value (0°) of *β*, a microcontroller (MCU) PIC24FV16KA304, a power supply module, and a signal acquisition circuit. Accordingly, a radio frequency (RF) transceiver (NRF24L01) was used in the Master unit to receive the stalk lodging angle *α* transmitted from the Slaver unit (Figure 2b), which consisted of another single axis angle sensor (SCA60C-N1000060) perpendicular to the circuit board of the Slaver unit with the initial value (0°) of *α*. In the Slaver unit, a single axis angle sensor (SCA60C-N1000060) and another RF transceiver (NRF24L01) were used to measure the stalk lodging angles in real time, which were later transmitted to the Master unit. In this measurement system, both high-performance microcontrollers, PIC24FV16KA301 and PIC24FV16KA304, were chosen with 12-bit high-precision analog/digital (A/D) converters, and they worked at the highest running speed of 16 million instructions per second (MIPS) to manage the measurement results. Furthermore, both single axis angle sensors were marked with arrows, indicating that both single axis angle sensors could measure angles ranging from −90° to +90° from the direction of the arrow. With this special structure, the values of the angle could be monitored in a changeable range from 0 to 90°, when the direction of the pull force was located above the horizontal plane. The instrumentation amplifier AD620 was utilized to amplify the voltage signals obtained from the strain sensor, due to the output’s voltage signals, in millivolts. The universal serial bus (USB) adapter chip CH340G was used to facilitate communication between the Master unit and a computer. The module BJ12864F was applied to display the measurement results, in 128 columns × 64 lines, in a 3-bit serial mode supplied by a unipolar voltage. By using this measurement system, the stalk lodging angle *α*, the pull force *F*, the equivalent force *F’*, and the angle *β* could be measured and displayed in real time.

### 2.3. Measurement Principle of Equivalent Forces

**Figure 3:**
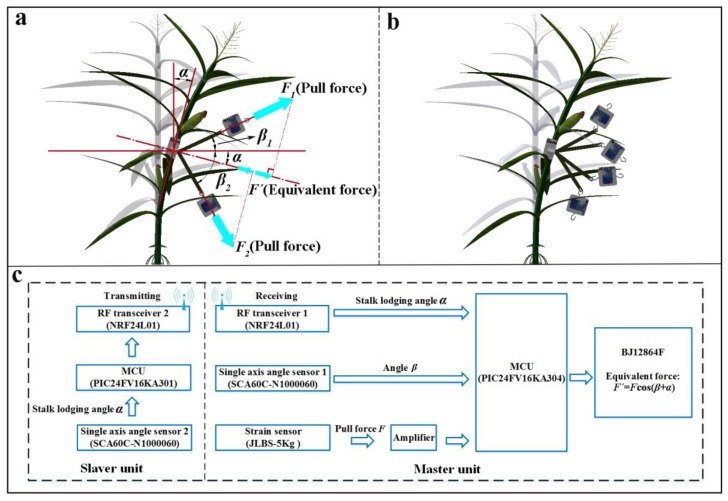
(**a**) Schematic diagram for the evaluation of stalk lodging properties under the pull force exerted upon the stalk; (**b**) some cases cannot occur during the experiment process; (**c**) the working schematics of the measurement system.

The schematic diagram of the measurement principle of the equivalent forces is shown in Figure 3. From Figure 3a, the pull force vector *F*_1_ or *F*_2_ can be decomposed into two parts: a downward part (or an upward part) along the corn stalk and a rightward part perpendicular to the corn stalk. The two vectors are independent of each other and have an influence upon the corn stalk in the bending process when the pull force is applied on the corn stalk. The sensing direction of the single axis angle sensors is marked with arrows on the surfaces of the Slaver and Master units, respectively. The angles ranging from −90° to +90° are reliably measured around the axis parallel to the sensing direction, where the angle reaches +90° when the arrow is standing up vertically to the horizontal plane and the angle is −90° when the arrow is standing in the opposite direction. The circuit board of the single axis angle sensor 1 was set to be perpendicular to the circuit board of the Master unit (Figure 2a) to ensure that the sensing direction of the single axis angle sensor 1 was always parallel to the horizontal plane at the initial position, when it was applied to measure the angles *β*_1_ or *β*_2_. In the case of an upward pull force *F*_1_ (shown in Figure 3a), the angle *β*_1_, measured by the single axis angle sensor mounted inside the Master unit, was positive, and the original pull force *F*_1_ was obtained by the strain sensor (JLBS-5Kg). The equivalent force *F*_1′_ was calculated using the following formula: *F*_1′_ = *F*_1_cos(*β*_1_ + *α*). However, the angle *β*_2_ was negative in the case of a downward pull force *F_2_* (shown in Figure 3a), and the equivalent force of *F*_2__′_ was also calculated by a similar formula: *F*_2′_ = *F*_2_cos(*β*_2_ + *α*)*.* Moreover, once the pull force was applied upon the inelastic belt through the operator, both the Master unit and Slaver unit should have been in a straight line, because the Slaver unit was connected to the Master unit with a non-rigid and inelastic belt. Therefore, those cases indicated in Figure 3b cannot occur in actual applications due to this special structure. In this experiment, the stalk lodging angles fluctuated within the range of 0° to 40° in the vertical plane. In order to evaluate the stalk lodging properties, an equivalent pull force was calculated by Equation (1) to eliminate the interference caused by the direction of the pull force. For standardization, the pull force was applied at the level of 440 mm along the corn stalk above the ground. Accordingly, the stalk lodging angle and the angles *β*_1_ or *β*_2_ were monitored at the same time (Figure 3a). For both cases shown in Figure 3a, the equivalent pull forces can be written in the following expression:

(1) $$F^{\prime }=F\cos(\beta +\alpha )$$

where *F* denotes the pull forces, *α* is a positive value measured by the Slaver unit whose direction is insensitive to the pull force due to the special design. In the case of an upward pull force *F*_1_ (Figure 3a), the measured angle *β* is also always positive. However, the measured angle *β* has a negative value under the action of a downward pull force *F*_2_ (Figure 3a). The equivalent force *F’* is consistently perpendicular to the cornstalk to eliminate the influence of the direction of pull force.

From the Figure 3c shown in the experiment process, the stalk lodging angle *α*, monitored by the single axis angle sensor 2 (SCA60C-N1000060), was transmitted by RF transceiver 2 (NRF24L01), which was mounted inside the Slaver unit, to RF transceiver 1 (NRF24L01) in the Master unit. The original pull force *F*, measured by the strain sensor (JLBS-5Kg), and angle *β*, obtained by the single axis angle sensor 1 (SCA60C-N1000060) mounted inside the Master unit, were processed by using the microcontroller (PIC24FV16KA304). The measurement results and equivalent forces were finally displayed on the liquid crystal display (LCD) screen (BJ12864F) in real-time.

## 3. Results and Discussion

### 3.1. Stability Results

**Figure 4:**
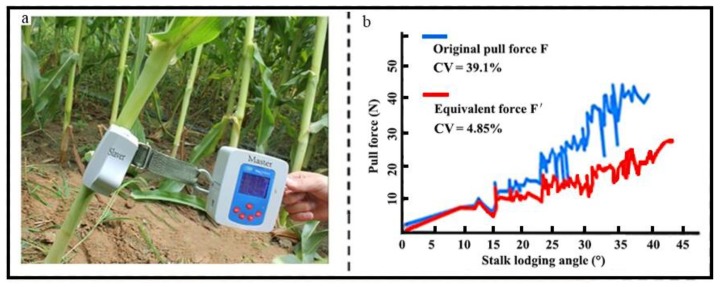
The pull force is exerted on a position of 440 mm above the ground and close to the fourth internode of the corn stalk. (**a**) The prototype of the measurement device applied in the field experiment of corn stalk lodging under the action of pull force *F*; (**b**) the original pull force *F* fluctuated significantly with a CV of 39.1% along with stalk lodging angles ranging from 0° to 45°, while the equivalent force was much more stable (CV of 4.85%).

Figure 4a depicts the measurement process performed by the novel measurement device, where the Slaver unit was fixed on a corn stalk with an inelastic belt, which was also connected to the strain sensor by a hook during the field measurement. After the Slaver unit and Master unit were well affixed, operators pulled the hook with a well-distributed force to avoid high dynamic tension in the measurement process. In the current measurement system, an acquisition circuit with the strain sensor had to be monitored with the weight of the Master unit enclosure (about 225 g) in real-time. The automatic zero-tracking calibration was run under normal operating conditions prior to applying the pull force on the stalk. To obtain adequate measurement data so as to clearly understand the stalk lodging process, the acquisition time of 0.1 s and the total measurement time of 20 s were set for each measurement. In this experiment, five different stalks from one of the corn varieties were used to conduct the measurement with the original pull force at different stalk lodging angles, *α*, which ranged from 0 to 45°. The coefficient of variations (CVs) of 39.1% and 4.85%, shown in Figure 4b, were obtained from the original pull force and the equivalent force, respectively. Compared with the CV of 39.1% obtained from the original pull force *F*, the measured equivalent force had a much lower CV of 4.85%, along with corn stalk lodging angles that ranged from 0° to 45°. These experimental results demonstrate that the equivalent force is less affected by the direction of the original pull force and has good stability in the corn stalk lodging process. The direction of the pull force largely depends on the height of the operators. Consequently, in the proposed method, the equivalent force, instead of the original pull force, is utilized to evaluate the properties of corn stalk lodging. This decreases the interference caused by the varying directions of the pull force exerted on the corn stalks.

### 3.2. Equivalent Forces Changed with Stalk Lodging Angles

**Figure 5:**
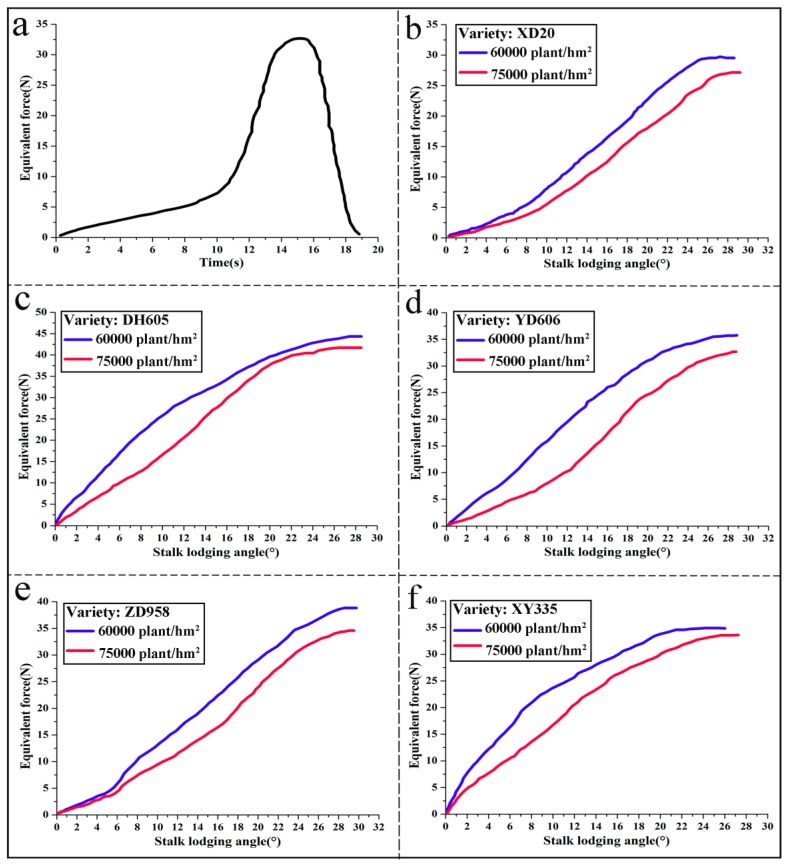
(**a**) Pull forces changing with the time in the process of corn stalk lodging; (**b**–**f**) the equivalent forces varying with corn stalk lodging angles for the five corn varieties with two planting densities, respectively.

By using the measurement device, the equivalent force and stalk lodging angle were monitored and stored continuously in flash memory, in real-time, during the corn stalk lodging process. In this field experiment, five different corn stalks from different positions in the center part of the plantation were chosen from each corn variety labeled Zhengdan958 (ZD958), Xianyu335 (XY335), Yudan606 (YD606), Xundan20 (XD20) and Denghai605 (DH605), with two planting densities of 60,000 plants/hm^2^ and 75,000 plants/hm^2^. The equivalent forces, varying with the stalk lodging angle *α*, were plotted in Figure 5, which were obtained from the results of five repeated measurements.

The typical lodging process against the measurement time *t*, under the pull force exerted upon the corn stalk, are illustrated in Figure 5a, while the equivalent forces, which varied with the stalk lodging angles obtained from five varieties of corn, are shown in Figure 5b–f, respectively. Obviously, it is noted that the maximum equivalent force from the planting density of 60,000 plants/hm^2^ is larger than that from the planting density of 75,000 plants/hm^2^. The measurement curves indicated in Figure 5 have non-smooth sections due to the presence of the uneven original pull force applied on the corn stalk, the non-uniform lodging speeds, or the jitter of original pull force that occurred during the process of stalk lodging. Conclusively, agricultural scientists could deduce the relationship between the stalk lodging angles and the equivalent forces during the process of corn stalk lodging from Figure 5.

### 3.3. Maximum Equivalent Forces

**Figure 6:**
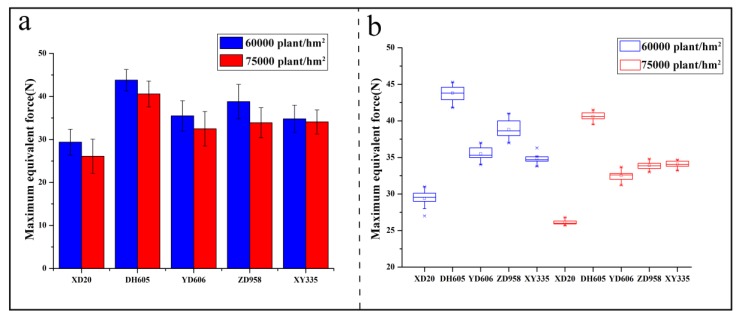
Comparison of the equivalent forces for the five corn varieties with two planting densities. (**a**) The maximum equivalent force measured by the measurement system for each variety of corn with different plant densities; (**b**) the box-plot for each variety of corn with different plant densities.

Five different corn stalks in the stage of silking (20 days before maturity), from each corn variety (ZD958, XY335, YD606, XD20, and DH605), with planting densities of 60,000 plants/hm^2^ and 75,000 plants/hm^2^, were measured from June to October in 2016 and 2017, respectively. The average measured results of the maximum equivalent forces were plotted in Figure 6a. 

Figure 6a indicates that the maximum equivalent forces obtained from both the different corn varieties and the same variety with different planting densities are quite different. From the pull forces applied upon the corn stalks from different planting densities, the maximum equivalent force is approximately 44 N for the planting density of 60,000 plants/hm^2^, and it is larger than that for the planting density of 75,000 plants/hm^2^ (Figure 6a).

Moreover, under the same planting density, the maximum equivalent force from DH605 is larger than the others. However, the equivalent forces obtained from ZD958 (75,000 plants/hm^2^), XY335, and YD606 are similar to each other. Summarily, the equivalent forces from the five varieties of corn with two planting densities of 75,000 plants/hm^2^ and 60,000 plants/hm^2^ show that the corn stalks from a high planting density area have less tensile strength than those from a low planting density area. The higher equivalent force of the corn stalk, the stronger lodging resistance the corn variety has. Figure 6b shows that the corn variety of DH605 (60,000 plants/hm^2^ and 75,000 plants/hm^2^) has a high maximum equivalent force, but the corn variety of XD20 (60,000 plants/hm^2^ and 75,000 plants/hm^2^) has a low maximum equivalent force.

### 3.4. Field Stalk Lodging Rates

**Figure 7:**
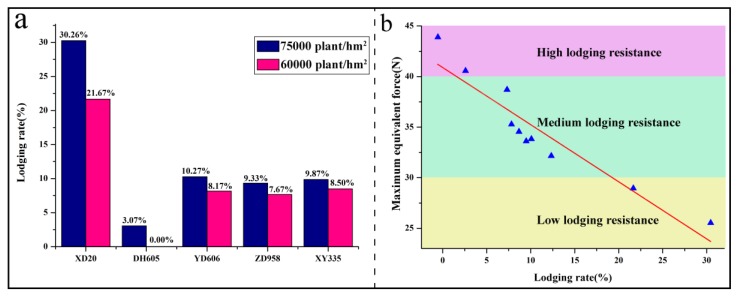
(**a**) The lodging rate calculated from the experiment samples; (**b**) the correlation between the maximum equivalent force and lodging rate.

The number of corn stalks that had already broken or lodged below the ear at harvest was counted for the five corn varieties grown in the Science and Education Farm of Henan Agriculture University, Zhengzhou, in 2016 and 2017. The average stalk lodging rate is presented in Figure 7a.

The results from the field survey show that the stalk lodging rate significantly increases with the planting density. The stalk lodging rate in the five varieties follows the order of DH605 (60,000 plants/hm^2^) < DH605 (75,000 plants/hm^2^) < ZD958 (60,000 plants/hm^2^) < YD606 (60,000 plants/hm^2^) < XY335 (60,000 plants/hm^2^) < ZD958 (75,000 plants/hm^2^) < XY335 (75,000 plants/hm^2^) < YD606 (75,000 plants/hm^2^) < XD20 (60,000 plants/hm^2^) < XD20 (75,000 plants/hm^2^). In comparison with the stalk lodging rate and the measurement results from the maximum equivalent forces, there is a strong correlation between the maximum equivalent force and the property of corn lodging resistance, and the square of the correlation coefficient of 0.88 was obtained between equivalent forces and field stalk lodging rates (Figure 7b). From Figure 7b, the corn variety of DH605, which has the maximum equivalent force larger than 40 N, possesses high lodging resistance, which was verified by the lower lodging rate (less than 5%). The maximum equivalent force obtained from the corn variety of XD20 is less than 30 N. Obviously, XD20 has low lodging resistance, for the most serious stalk lodging rate (larger than 20%) happened to this variety. Correspondingly, lodging rates obtained from the rest of the corn varieties, such as ZD958, XY335, and YD606, are in the range of 5% to 20%. This clearly indicates that these varieties, with maximum equivalent forces ranging from 30 N to 40 N, have medium lodging resistance.

## 4. Conclusions

This study demonstrated a non-destructive and direction-insensitive method to evaluate the stalk lodging properties of corn by their measured equivalent forces. The novel measurement device used includes both a Slaver unit and a Master unit, which mainly consists of a strain sensor, two single axis angle sensors, and two RF transceivers (NRF24L01). Five varieties of corn, labeled Zhengdan958 (ZD958), Xianyu335 (XY335), Yudan606 (YD606), Xundan20 (XD20), and Denghai605 (DH605), were grown in the plant distribution, at the planting densities of 60,000 plants/hm^2^ and 75,000 plants/hm^2^, to verify the performance of this measurement method. A corn stalk has a high lodging resistance if the average maximum measured equivalent force is larger than 40 N, whereas a corn stalk has a medium lodging resistance if the average measured maximum equivalent force is in the range of 30 N to 40 N. Accordingly, for those corn varieties, a stalk has a low lodging resistance if the average maximum measured equivalent force is less than 30 N. In conclusion, the experimental results validate that this non-destructive and direction-insensitive measurement method could have a great potential to evaluate the properties of corn stalk lodging in real-time in the field. In future work, more corn varieties could be studied both to improve the analysis accuracy and to make the evaluation results more reliable. 

## Figures and Tables

**Table 1:** Agronomic traits obtained from five varieties of corn with two planting densities.

Sample	Density (Plants/hm^2^)	Variety	Plant Height (*H_p_*)/mm	Ear Height (*H_e_*)/mm	Stalk Diameter (*D_S_*)/mm	Internode Length (*L_i_*)/mm	Ear Height Coefficient (*Ce*)
1	60,000	ZD958	2606.00 b	1080.00 b	23.10 a	71.00 c	0.40 bc
2	60,000	YD606	2603.00 b	1060.20 c	23.20 a	98.10 a	0.41 b
3	60,000	XD20	2464.20 d	1183.10 a	23.40 a	94.00 ab	0.48 a
4	60,000	XY335	2702.20 a	1093.10 b	23.10 a	93.00 b	0.41 b
5	60,000	DH605	2538.00 c	1000.00 c	23.40 a	74.20 c	0.39 c
6	75,000	ZD958	2620.10 b	1106.00 c	22.50 a	80.00 c	0.42 b
7	75,000	YD606	2652.00 b	1076.20 c	22.11 a	109.30 ab	0.42 b
8	75,000	XD20	2547.40 c	1272.00 a	21.60 a	111.00 a	0.50 a
9	75,000	XY335	2736.30 a	1140.60 b	22.50 a	95.10 b	0.42 b
10	75,000	DH605	2562.00 c	1030.60 c	22.60 a	78.20 c	0.40 c

Note: The lowercase letters a, b, c and d in Table 1 indicate the significant differences between different corn varieties for each stalk’s agronomic traits, including plant height (*H_p_*), ear height (*H_e_*), stalk diameter (*D_s_*), internode length (*L_i_*), and ear position coefficient (*C_e_*), which were achieved by using the Least Significant Difference (LSD) (the *p*-value < 0.05). Here, the plant height (*H_p_*) is the distance from root surface to tip of tassels. *H_e_* is the distance from root surface to node of top ear. The stalk diameter (*D_s_*) at mid-growth stages is measured by using a Vernier caliper. The internode length (*L_i_*) is the average internode length of the stalk between the 3rd node and 4th node counting from the ground. The ear position coefficient (*C_e_*) is the ratio of the ear height to the plant height.
